# Supplementary material for: The local stability of a modified multi-strain SIR model for emerging viral strains
Source: PLoS One. 2020 Dec 9;15(12):e0243408. doi: 10.1371/journal.pone.0243408 (PMC7725381; doi:10.1371/journal.pone.0243408)
Supplement: S1 Table — (PDF) [file pone.0243408.s001.pdf]

S1 Table. List of variables used in describing the emergent strain model.

**Symbol**	**Definition**
μ	Birth/Death rate
p	Vaccination rate
β	Standard incidence transmission coefficient
γ	Removal rate coefficient
β'	Standard incidence transmission coefficient (emergent strain)
γ'	Removal rate coefficient (emergent strain)
N	Total population number
S	Number of susceptible individuals
s	Proportion of susceptible individuals
V	Number of vaccinated individuals
v	Proportion of vaccinated individuals
I_1	Number of individuals infected by the original strain
i_1	Proportion of individuals infected by the original strain
I_2	Number of individuals infected by the emergent strain
i_2	Proportion of individuals infected by the emergent strain
R_1	Number of individuals recovered from the original strain
r_1	Proportion of individuals infected by the original strain
R_2	Number of individuals removed after getting infected by the emergent strain
r_2	Proportion of individuals removed after getting infected by the emergent strain
